# Effect of *Ficus glomerata* leaf extract in streptozotocin-induced early diabetic complications and its characterization by LC-MS

**DOI:** 10.17179/excli2019-1441

**Published:** 2020-01-03

**Authors:** Abusufyan Shaikh, Mohammed Ibrahim, Mohib Khan

**Affiliations:** 1 School of Pharmacy, Anjuman-I-Islam’s Kalsekar Technical Campus, New Panvel, Maharashtra, affiliated to Mumbai University, Mumbai, India; 2 Research Scholar, JNTUH, Kukatpally, Telangana, India; 3 PNR College of Pharmacy, Telangana, India; 4 Oriental College of Pharmacy, Navi Mumbai, India

**Keywords:** antidiabetic activity, antioxidant activity, Ficus glomerata, phytoconstituents, streptozotocin

## Abstract

Diabetes mellitus is a complex metabolic disorder that leads to various micro-vascular complications. The present study elucidated the effect of chloroform extract of leaves of *Ficus glomerata* (CHFG) in streptozotocin-induced early diabetic renal and neural complications. Wistar rats were injected with STZ (55 mg/kg, i.p.) to produce experimental diabetes. Two weeks after the stabilization of diabetes, CHFG extract at the dose of 200 and 400 mg/kg (CHFG 200 and CHFG 400) and metformin at the dose of 250 mg/kg (Met 250) was administered to the diabetic rats for further two weeks. Diabetic rats showed an increase in blood glucose, plasma urea, uric acid, creatinine, triglyceride, and total cholesterol level. The change in behavioral parameters such as thermal hyperalgesia and cold allodynia with compromised sciatic nerve and kidney antioxidant status were seen in diabetic rats. Diabetic rats treated with CHFG 200, CHFG 400, and Met 250 showed a decrease in blood glucose, plasma urea, uric acid, creatinine, triglyceride, and total cholesterol level. Also, it improved altered behavioral parameters such as thermal hyperalgesia and cold allodynia. It also restored the sciatic nerve and kidney antioxidant status. The results of kidney and sciatic nerves histopathological study were in line with the results of biochemical parameters that confirmed the favorable role of CHFG. Characterization of CHFG by LC-MS revealed the presence of diverse phytoconstituents, which might be responsible for its protective effect.

## Introduction

Diabetes mellitus (DM) is a complicated disorder caused by genetic or environmental factors (Bronwyn et al., 1995[11]; Fatemeh et al., 2009[18]; Todd et al., 1987[51]). It is one of the most common endocrine diseases with its global prevalence of 9.3 % of the world population and is predicted to rise to 10.2 % by 2030 and 10.9 % by 2045 (International Diabetes Federation, 2019[21]). All forms of diabetes are characterized by chronic hyperglycemia and the development of diabetes-specific microvascular complications (Ohtsuji et al., 2008[40]). As a result of this, diabetes is one of the major cause of neuropathic pain, end-stage renal disease, and cardiovascular diseases. 

Chronic hyperglycemia stimulates the polyol pathway, causing osmotic stress, increases the generation of reactive oxygen species, which leads to oxidative stress in the tissues. An increasing body of data supports the role of oxidative stress in the pathogenesis of diabetic microvascular complications (Ahmad et al., 2015[3]; Bashan et al., 2009[7]; Mahmood and Neaimy, 2008[31]; Nishikawa et al., 2000[39]). There are many drugs to treat diabetes, but none have been successful for the treatments of its complications completely. Therefore its treatment remained as one of the challenge faced by modern medicine. Hyperglycemic control remains a primary therapeutic target when dealing with diabetic complications in both types of diabetes (Mahmood and Neaimy, 2008[31]; Sima and Kamiya, 2006[48]). A significant benefit of the use of phytoconstituents, minerals, and vitamins is thought to be due to its capability to scavenge free radicals and thus lowering the incidence of diabetic complications (Pirat, 1977[43]; Dal and Sigrist, 2016[13]). Plants are always a good source of active pharmacological agents, and many of the currently available therapeutic agents have been derived from them (Patel et al., 2012[41]). Many biologically active compounds from plant sources such as phenolics, flavonoids, lycopene, saponins, triterpenoids, and many others have the antidiabetic effect (Grover et al., 2002[20]). Traditional, complementary, and alternative medicines are increasingly used for the treatment of diabetes and its complications (Dodda and Ciddi, 2014[16]; Snehal et al., 2017[49]; Dal and Sigrist, 2016[13]). 

Various medicinal plants, especially those belonging to the genus *Ficus* and its phytoconstituents, have been used for the treatment of diabetes and related chronic complications since ancient times (Deepa et al., 2018[15]). *Ficus glomerata *(FG), family *Moraceae* is well known as a Cluster fig tree, Goolar, Indian fig tree, Umber, Udumbara, Rumadi, Dimri, Blue lotus found in the Indian subcontinent, Indo-China, Australia and Malaysia (Joy et al., 2001[23]; Kunwar and Bussmann, 2006[28]). All parts of FG are medicinally important and used widely in the treatment of jaundice, wound healing, dysentery, diabetes, biliary disorders and inflammatory conditions (Nadkarni, 1976[37]; Patil et al., 2009[42]; Satish et al., 2014[46]). Our previous study revealed the *in vitro* antidiabetic and antioxidant activity of CHFG (Abusufyan et al., 2018[1]). However, there is no *in vivo *data about the efficacy of this extract available in diabetic renal and neural complications. Based on this, CHFG was selected to investigate its effect on early diabetic renal and neural complications.

## Materials and Methods

### Chemicals reagents

A free sample of metformin procured from USV Pvt Ltd, Mumbai. Streptozotocin was purchased from Sisco Research Laboratories Pvt. Ltd, Mumbai. All other chemicals were of analytical grade and obtained from commercial sources.

### Preparation and selection of FG leaf extract

The leaves of FG were collected from the Sindhudurg district of Maharashtra, India, and identified with authentication no. 15-023 by botanist Dr. A. S. Upadhye of Agharkar Research Institute, Pune, India. The powdered leaf material was extracted by successive solvent extraction with the solvents of increasing order of polarity and concentrated by using a rotary evaporator. Based on our previous study, chloroform extract (CHFG) was selected for the final investigation as it showed maximum antidiabetic and considerable antioxidant activity (Abusufyan et al., 2018[1]).

### CHFG extract analysis

The preliminary phytochemical estimation of the extracts was studied by the standard method of Khandelwal (2013[26]). The HR-LCMS of the selected extract was carried out at the Sophisticated Analytical Instrument Facility (SAIF) of IIT Bombay, Pawai, Mumbai, by using Agilent high-resolution liquid chromatography and mass spectrometry model- G6550A with 0.01 % mass resolution. The electrospray ion source was used to operate the MS system. Solvents used were 100 % of water (A) and 100 % of acetonitrile (B). The elution started with a linear gradient; within 30 minutes of acquisition time, the initial 2 minutes, the flow of solvent composition A: B was 95:5. The injection volume was 3 µl, and the flow rate was 0.2 ml min^-1^. The MS signals were used for qualitative analysis based on specific mass spectra. The chromatographic data were analyzed by using the software from Agilent, USA. For the detection of compounds, the MS spectra of the analyzed samples were compared with the spectra from the library. 

### Acute oral toxicity test 

Acute oral toxicity test was conducted as per the OECD guideline No. 423. A single oral dose of 2000 mg/kg of CHFG was administered to overnight fasting Wistar albino rats. The sign and symptoms of toxicity were observed in the animals for 24 hours. The mortality, if any, was noted for two weeks.

### Oral glucose tolerance test (OGTT) and selection of dose

Doses equivalent to 200, 400, and 600 mg/kg of CHFG extract were calculated and suspended in 1 % v/v of tween 80 solutions. OGTT was carried out on overnight fasting normal rats. 1 % v/v of tween 80, CHFG (200, 400, and 600 mg/kg) and metformin (250 mg/kg) were administered to five groups of rats, respectively. Glucose (2 g/kg) was fed 30 min after pre-treatment with 1 % v/v of tween 80, CHFG, and metformin. Blood glucose levels were observed at 0, 30, 60, 120, and 240 min after glucose load to assess the effect of the extract. Two doses with optimum dose-dependent anti-hyperglycemic activity against glucose tolerance in normal rats were selected for the final study in STZ induced diabetic rats.

### Animal's treatments and experimental design

Healthy male Wistar albino rats weighing 300-350 g were procured from the National Institute of Biosciences, Pune, Maharashtra, India, and housed in polypropylene cages at the temperature of 22 ± 1 °C and relative humidity of 50 to 60 % with a 12 h light/dark cycle. The animal experiments were approved by the Institutional Animal Ethics Committee (IAEC), School of Pharmacy-AIKTC, New Panvel (Approval No. AIKTC/SoP/IAEC/ Approval/2016/1). Standard pellet diet and water ad libitum were provided to the experimental animals throughout the experiment. Diabetes was induced by a single injection of streptozotocin (STZ, 55 mg/kg, i.p.) freshly prepared in 0.1 M citrate buffer pH 4.5) in rats (Azahar et al., 2012[6]). Blood glucose level was measured, and rats with blood glucose levels above 250 mg/dl were considered diabetic. Two weeks post-STZ injection diabetic rats with stable blood glucose level were divided into different groups as follow:

*Group* *I:* Normal control (n = 6), normal rats treated with 10 ml/kg, p.o. of distilled water.

*Group II:* Diabetic control (n = 6), STZ induced diabetic rats treated with vehicle (1 % v/v of tween 80 in distilled water 10 ml/kg, p.o.).

*Group III:* STZ + Met 250 (n = 6), STZ induced diabetic rats treated with Metformin 250 mg/kg. 

*Group IV:* STZ + CHFG 200 (n = 6), STZ induced diabetic rats treated with CHFG 200 mg/kg. 

*Group V:* STZ + CHFG 400 (n = 6), STZ induced diabetic rats treated with CHFG 400 mg/kg.

All the treatments were administered daily via a standard orogastric cannula for two weeks. The changes in the blood glucose level, body weight, thermal hyperalgesia, and cold allodynia were observed after one and two weeks post-treatment. At the end of the treatment, 12 hours fasted rats were euthanized, and blood samples were collected by the retro-orbital plexus. The blood samples were centrifuged at 1300 g for separation of serum and used for estimation of serum urea, uric acid, creatinine, triglycerides, and total cholesterol level. Kidneys and sciatic nerves were isolated from euthanized animals, rinsed with ice-cold saline, and used for estimation of tissue antioxidant status and histopathological study.

### Measurements of fasting blood glucose 

The fasting blood glucose was measured to study the effect of pre-treatment, one and two-week post-treatment on lateral tail vein blood samples using a OneTouch glucometer (Lifescan Scotland Ltd) throughout the treatment period.

### Behavioral assessment of early diabetic neuropathy

Both thermal hyperalgesia and cold allodynia were studied after one and two-week post-treatment. Thermal hyperalgesia was studied by measuring paw and tail withdrawal latency (sec) by hot plate and tail immersion test at 49 ± 0.5 ^o^C. Shortening of the paw and tail withdrawal latency indicates thermal hyperalgesia. Cold allodynia was studied by measuring tail withdrawal latency (sec) by dipping the tail in the water at a cold temp (10 ± 0.5 °C), which is usually innocuous. 

### Determination of the serum biochemical parameters

Serum urea, uric acid, creatinine, triglycerides, and total cholesterol levels were measured by using the standard commercially available kits and as per the instructions of manufacturer's.

### Determination of tissue antioxidant status

At the end of the experiment, the sciatic nerve and kidney were isolated and washed with ice-cold saline. Then homogenate was made and used for determination of MDA, an end product of lipid peroxidation (Mihara and Uchiyama, 1978[34]), reduced glutathione (GSH) (Ellman, 1959[17]), and superoxide dismutase (SOD) (Misra and Fridovich, 1972[35]).

### Histopathological examinations

The part of the isolated kidney and sciatic nerves were fixed in 10 % formalin. The paraffin embedded sections of 5 μm thickness were cut and stained with hematoxylin and eosin (H&E). The kidney histological sections were examined by light microscopy at 20x, whereas the sciatic nerve sections were examined by oil immersion lens (100x). Quantitative estimation of urinary space, glomerular tuft area (GA) of kidney sections, mean axonal, and fiber diameter in sciatic nerve sections was determined by using ImageJ software. The glomerular volume (GV) was calculated by converting GA to GV by applying a spherical approximation formula (GV = 1.2545 (GA)^1.5^) (Gopala and Greg, 2007[19]). For each parameter, six images from six different samples of each study group were analyzed. Five non-overlapping fields from each image were captured. Five different readings from every captured photo were counted, and the mean was calculated for each image. 

### Data analysis and statistics

One-way analysis of variance (ANOVA) followed by post-hoc Tukey's HSD test was performed to conduct statistical analysis. All values were expressed as means ± SEM. Differences were considered significant when *P < 0.05 and highly significant when **P < 0.01.

## Results

### Preliminary phytochemical analysis and LC-MS characterization of CHFG 

Preliminary phytochemical estimation revealed the presence of alkaloids, flavonoids, glycosides, phenolics, and saponins. CHFG was characterized by LC-MS, and phytoconstituents in the extract were identified by the compound library. The list of the compounds detected is given in Table 1(Tab. 1). 

### Acute oral toxicity study 

In the present study, none of the rats showed visible signs of toxicity after treatment with a single oral dose of 2000 mg/kg of CHFG extract. Observations twice daily for two weeks also did not reveal any drug-related toxicity.

### Effect on oral glucose tolerance test and selection of dose

**Figure 1:**
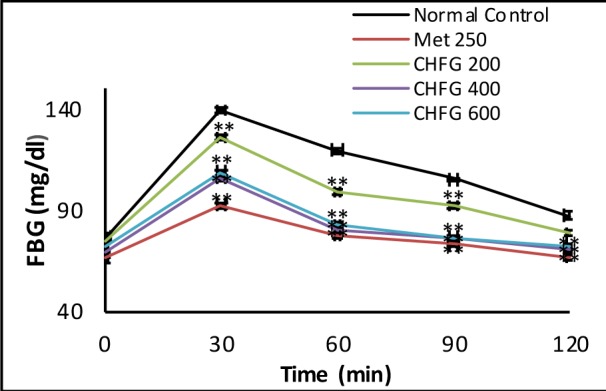
Effect of single-dose treatment on oral glucose tolerance in normoglycemic rats. All values were expressed as Mean + SEM, (n = 6); *P < 0.05, **P < 0.01 as compared to normal control group.

Hypoglycemic activity of CHFG 200, CHFG 400, and CHFG 600 of extract and Met 250 on glucose tolerance was examined in glucose loaded rats (Figure 1(Fig. 1)). Rats treated with CHFG 200, CHFG 400, and CHFG 600 and Met 250 mg/kg significantly (P < 0.01) lower the FBG level in comparison to the normal control group. The dose-dependent decrease in FBG level was observed in CHFG 200 and CHFG 400. However, CHFG 600 fails to show a significant dose-dependent activity as compared to its lower dose of CHFG 400. Hence, for the final study in STZ induced diabetic rats, two doses, CHFG 200 and CHFG 400, were selected.

### Effect on fasting blood glucose level

**Figure 2:**
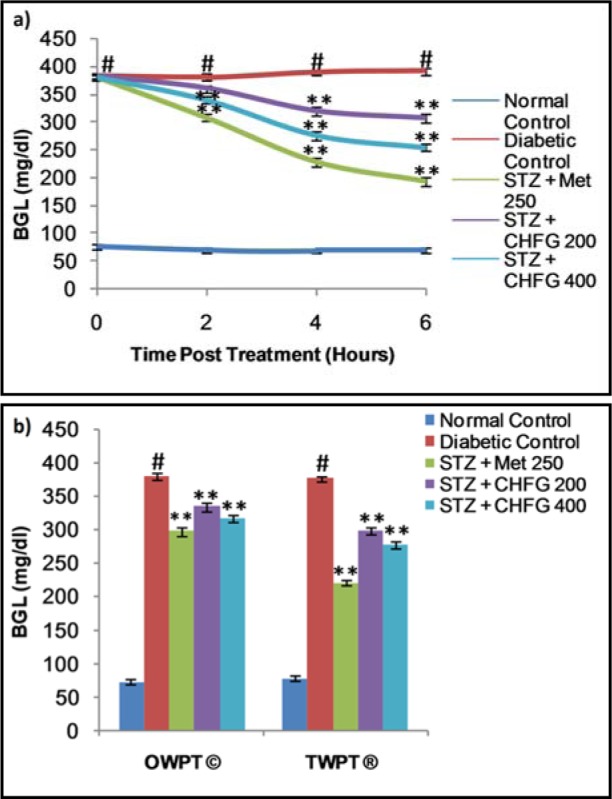
a) Effect of single-dose treatment on blood glucose level in STZ-induced diabetic rats. b) Effect of repeated-dose treatment on blood glucose level in STZ-induced diabetic rats. OWPT: One Week Post Treatment, © = observation recorded three weeks after induction of diabetes, TWPT: Two Weeks Post Treatment, ® = observation recorded four weeks after induction of diabetes, All values were expressed as Mean + SEM, (n = 6); #P < 0.01, as compared to normal control group; *P < 0.05, **P < 0.01 as compared to diabetic control group.

The fasting blood glucose levels for both single and repeated dose treatment with Met 250, CHFG 200, and CHFG 400 were measured in STZ-induced diabetic rats (Figure 2a and 2b(Fig. 2)). Fasting blood glucose level of single-dose treatment was fall significantly (P < 0.01) after 4 and 6 hours post-treatment in Met 250, CHFG 200, and CHFG 400 group as compared to the diabetic control group (Figure 2a(Fig. 2)). There were 41.27 %, 17.88 %, and 29.13 % fall in blood glucose level after 4 hours post-treatment in Met 250, CHFG 200, and CHFG 400 groups, respectively, as compared to the diabetic control group. There was 50.53 %, 21.64 %, and 34.98 % fall in blood glucose level after 6 hours post-treatment in Met 250, CHFG 200, and CHFG 400 groups, respectively, as compared to the diabetic control group.

Repeated dose treatment with Met 250, CHFG 200, and CHFG 400 showed significant (P < 0.01) fall in blood glucose level compared to the diabetic control group (Figure 2b(Fig. 2)). There was 21.64 %, 11.12 %, and 16.64 % fall in fasting blood glucose level after one-week post-treatment (OWPT) in Met 250, CHFG 200, and CHFG 400 groups respectively as compared to the diabetic control group. There were 41.29 %, 20.58 %, and 26.21 % fall in fasting blood glucose level after two weeks post-treatment (TWPT) in Met 250, CHFG 200, and CHFG 400 groups respectively as compared to the diabetic control group. 

### Effect on thermal hyperalgesia

**Figure 3:**
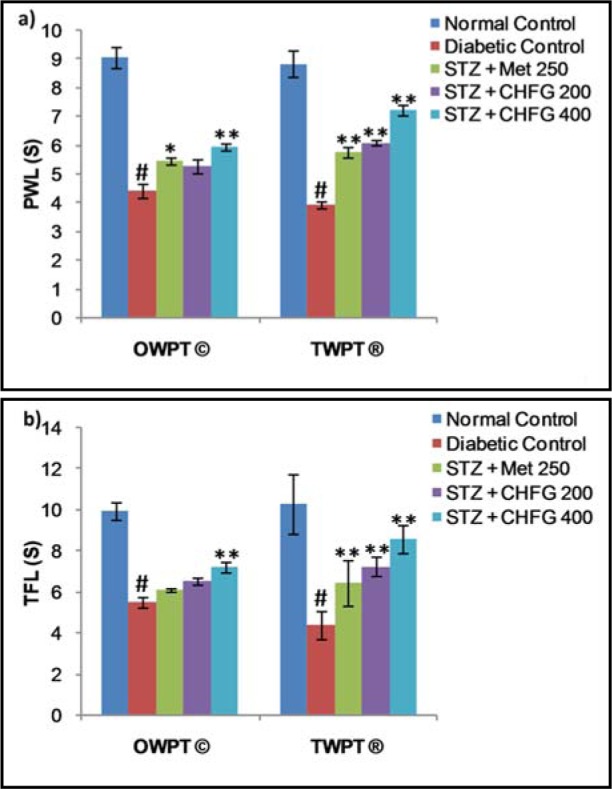
a) Effect of repeated-dose treatment on paw withdrawal latency (PWL) using a hot plate test at 49 °C in STZ-induced diabetic rats. b) Effect of repeated-dose treatment on tail-flick latency (TFL) using water immersion test at 49 °C in STZ-induced diabetic rats. OWPT: One Week Post Treatment, © = observation recorded three weeks after induction of diabetes, TWPT: Two Weeks Post Treatment, ® = observation recorded four weeks after induction of diabetes, All values were expressed as Mean + SEM, (n = 6); # P < 0.01, as compared to normal control group; *P < 0.05, **P < 0.01 as compared to diabetic control group.

The pain threshold was significantly reduced in diabetic rats as compared with the normal control group (Figure 3a and 3b(Fig. 3)). Treatment of diabetic rats with Met 250, CHFG 200, and CHFG 400 revealed an increase in pain threshold after OWPT and TWPT as compared to the diabetic control group.

### Effect on cold allodynia

**Figure 4:**
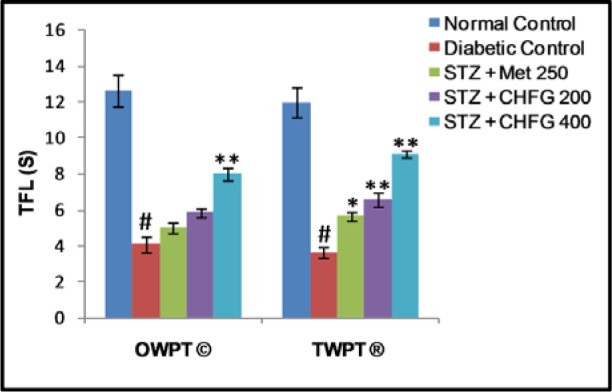
Effect of repeated-dose treatment on cold allodynia using water immersion test at 10 °C in STZ-induced diabetic rats. OWPT: One Week Post Treatment, © = observation recorded three weeks after induction of diabetes, TWPT: Two Weeks Post Treatment, ® = observation recorded four weeks after induction of diabetes, All values were expressed as Mean + SEM, (n = 6); #P < 0.01, as compared to normal control group; *P < 0.05, **P < 0.01 as compared to diabetic control group.

Tail flick latency at 10 °C was significantly (P < 0.01) lower in diabetic rats after two weeks of diabetes as compared with the normal control group (Figure 4(Fig. 4)). Treatment of diabetic rats with CHFG 400 showed statistically significant (P < 0.01) increase in pain threshold after OWPT and TWPT as compared to the diabetic control group. Although Met 250 and CHFG 200 fails to show a statistically significant difference in OWPT but showed a statistically significant increase in pain threshold in Met 250 (P < 0.05), CHFG 200 (P < 0.01) after TWPT as compared to the diabetic control group.

### Effect on body weight, kidney weight and kidney weight to body weight ratio

**Figure 5:**
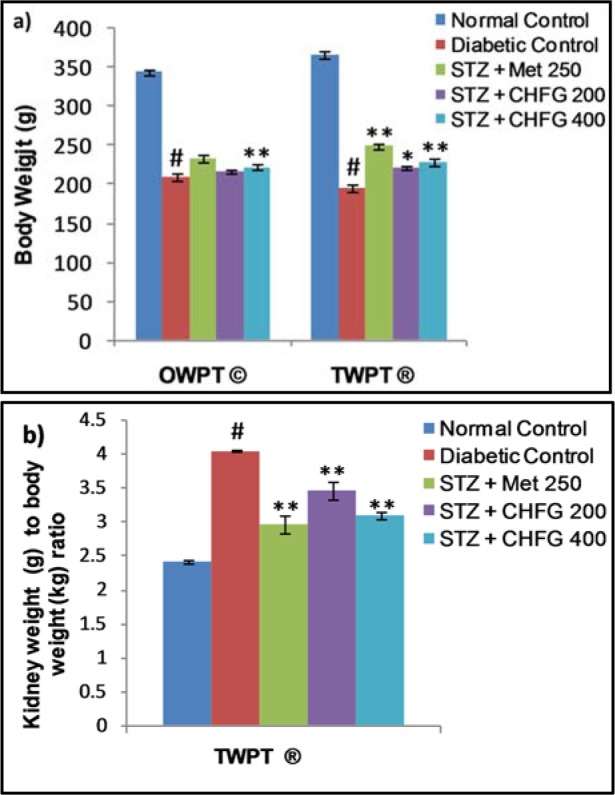
a) Effect of repeated-dose treatment on body weight in STZ-induced diabetic rats. b) Effect of repeated-dose treatment on kidney weight to body weight ratio in STZ-induced diabetic rats. OWPT: One Week Post Treatment, © = observation recorded three weeks after induction of diabetes, TWPT: Two Weeks Post Treatment, ® = observation recorded four weeks after induction of diabetes, All values were expressed as Mean + SEM, (n = 6); #P < 0.01, as compared to normal control group; *P < 0.05, **P < 0.01 as compared to diabetic control group.

A statistically significant (P < 0.01) decrease in body weight in the diabetic control group was observed as compared to the normal control group. OWPT treatment with CHFG 400 showed a statistically significant (P < 0.01) increase in the body weight as compared to the diabetic control group. Considerable increase in the body weight after TWPT with Met 250 (P < 0.01), CHFG 200 (P < 0.05), and CHFG 400 (P < 0.01) was observed against the diabetic control group (Figure 5a(Fig. 5)). Moreover, there was a remarkable elevation in kidney weights to body weight ratio in the diabetic control group. In contrast, kidney weight to body weight ratio was significantly (P < 0.01) lower in the TWPT with Met 250, CHFG 200 and CHFG 400 treatment group as compared to the diabetic control group (Figure 5b(Fig. 5)).

### Effect on biochemical parameters

Table 2(Tab. 2) presents the study on serum urea, uric acid, creatinine, triglycerides, and total cholesterol level. In comparison to the normal control group, STZ treated diabetic rats showed a significant (P < 0.01) increase in serum urea, uric acid, creatinine, triglyceride, and total cholesterol level. Met 250, CHFG 200, and CHFG 400 showed a significant (P < 0.01) decrease in serum urea level as compared to the diabetic control group. TWPT with Met 250 and CHFG 400 significantly (P < 0.01) reduced serum uric acid, creatinine, triglyceride, and total cholesterol level as compared to the diabetic control group. TWPT with CHFG 200 significantly (P < 0.05) decreased serum triglyceride, but it fails to show a significant change in serum creatinine and total cholesterol level as compared to the diabetic control group.

### Effect on serum antioxidant status

Significant (P < 0.01) increase in MDA level (3.08 fold in sciatic nerve and 1.79 fold in kidney tissue), decrease in GSH (1.73 fold in sciatic nerve and 1.96 fold in kidney tissue) and decrease in SOD (1.66 fold in sciatic nerve and 1.85 fold in kidney tissue) were observed in diabetic control rats as compared to normal control rats (Table 3(Tab. 3)). 

Administration of Met 250 for two weeks significantly (P < 0.01) decreases MDA (2.02 fold in sciatic nerve and 1.44 fold in kidney tissue), increases GSH (1.47 fold in sciatic nerve and 1.46 fold in kidney tissue) and increases SOD (1.36 fold in sciatic nerve and 1.53 fold in kidney tissue) as compared to their level in respective tissues in diabetic control group (Table 3(Tab. 3)).

TWPT with CHFG 200 showed a significant decrease in MDA level (1.58 fold in the sciatic nerve and 1.27 fold in kidney tissue) as compared to their level in respective tissues in the diabetic control group. Although TWPT with CHFG 200 increases GSH level by 1.21 fold in the sciatic nerve and 1.27 fold in kidney tissue but this improvement was found to be non-significant as compared to their level in respective tissues in the diabetic control group. Also, CHFG 200 showed a significant increase in SOD by 1.25 fold in sciatic nerve (P < 0.01) and 1.28 fold in kidney tissue (P-ns) as compared to their level in respective tissues in the diabetic control group (Table 3(Tab. 3)). Whereas treatment with CHFG 400 for two weeks significantly (P < 0.01) decreases MDA (2.43 fold in sciatic nerve and 1.66 fold in kidney tissue), increases (P < 0.01) GSH (1.58 fold in sciatic nerve and 1.76 fold in kidney tissue) and increases (P < 0.01) SOD (1.41 fold in sciatic nerve and 1.59 fold in kidney tissue) as compared to their level in respective tissues in diabetic control group (Table 3(Tab. 3)).

### Effect of CHFG on histopathological analysis of kidney and sciatic nerve

**Figure 6:**
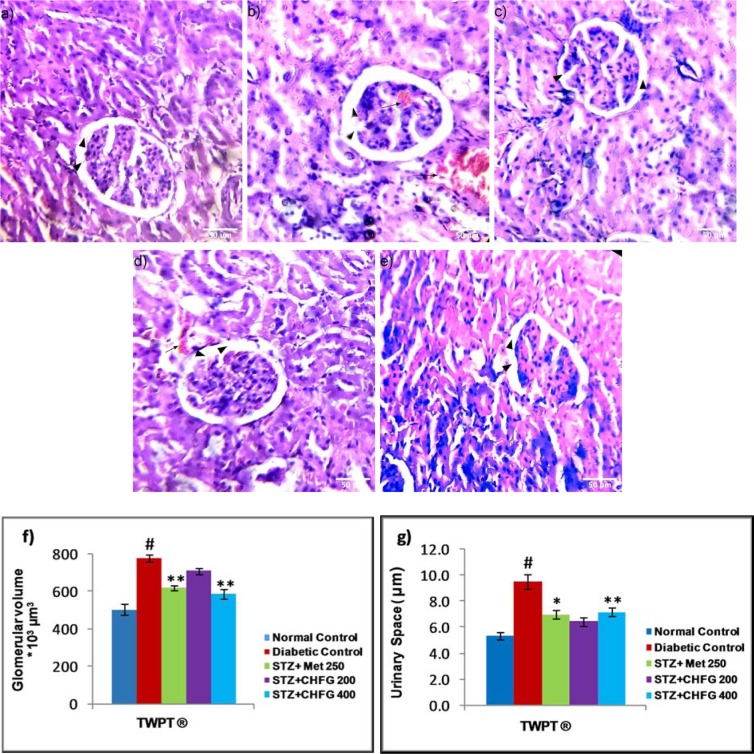
Light microscopic representative images of kidney tissues (HE stained sections, 20X, Scale bar 50 μm). a) Normal control, showing normal urinary space, and glomerular volume (arrowhead). b) Diabetic control, showing an increase in urinary space (arrowhead), increased in glomerular tuft area (arrowhead) and the presence of capillaries congestion with hemorrhage (arrow) as compared to normal control. c) STZ + Met 250 showed a decrease in urinary space (arrowhead), decreased in glomerular tuft area (arrowhead), and the absence of capillaries congestion without hemorrhage. d) STZ + CHFG 200 showed an insignificant decrease in urinary space (arrowhead), decreased in glomerular tuft area (arrowhead), and evidence of hemorrhage (arrow). e) STZ + CHFG 400 showed a decrease in urinary space (arrowhead), decreased in glomerular tuft area (arrowhead), and absence of capillaries congestion with no evidence of hemorrhage. f) Effect of repeated-dose treatment on the glomerular volume of kidney tissue. g) Effect of repeated-dose treatment on urinary space of kidney tissue. TWPT: Two Weeks Post Treatment, ® = observation recorded four weeks after induction of diabetes. All values were expressed as Mean + SEM, (n = 6); #P < 0.01, as compared to the normal control group; *P < 0.05, **P < 0.01 as compared to the diabetic control group.

Figure 6(Fig. 6) showed the effect of *CHFG* on glomerular morphology. The histopathological study revealed normal glomeruli in the normal control group (Figure 6a(Fig. 6)). The kidney sections of diabetic rats showed significant (P < 0.01) increase in glomerular volume and urinary space (P < 0.01), and presence of glomerular capillary congestion with hemorrhage as compared to kidney section of the normal control group (Figure 6b, f, and g(Fig. 6)). The TWPT with Met 250 and CHFG 400 showed statistically significant (P < 0.01) decrease in glomerular volume and urinary space (P < 0.05 in Met 250, and P < 0.01 in CHFG 400) and absence of capillaries congestion without hemorrhage (Figure 6c, e, f, and g(Fig. 6)). Whereas, the TWPT with CHFG 200 showed statistically insignificant changes in glomerular volume and urinary space as compared to the diabetic control group. Also, it showed the evidence of hemorrhage with the minor blood-filled area (Figure 6d, f, and g(Fig. 6)).

**Figure 7:**
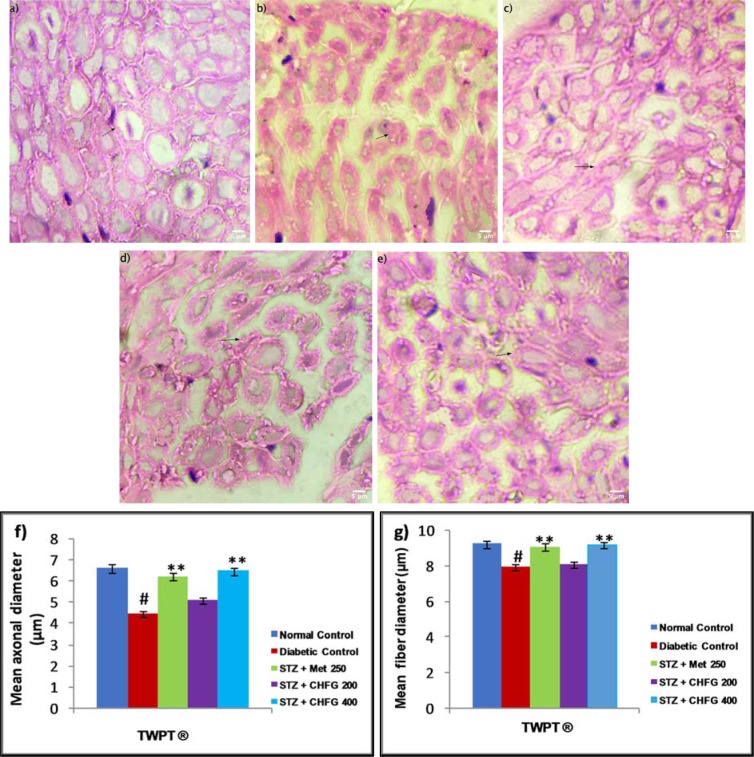
Light microscopic representative images of sciatic nerve tissue (HE stained sections, 100X, immersion oil, Scale bar 5 μm). a) Normal control showing normal histological features of axonal fiber (arrow). b) Diabetic control showing axonal atrophy (arrow). c) STZ + Met 250 showing less axonal atrophy as compared to the diabetic control group. d) STZ + CHFG 200 showed axonal atrophy (arrow). e) STZ + CHFG 400, showing less axonal atrophy as compared to the diabetic control group. f) Effect of repeated-dose treatment on the mean axonal diameter of sciatic nerve tissue. g) Effect of repeated-dose treatment on the mean fiber diameter of sciatic nerve tissue. TWPT: Two Weeks Post Treatment, ® = observation recorded four weeks after induction of diabetes. All values were expressed as Mean + SEM, (n = 6); #P < 0.01, as compared to the normal control group; *P < 0.05, **P < 0.01 as compared to the diabetic control group.

Histopathological study of the normal control group showed normal sciatic nerve architecture (Figure 7a(Fig. 7)). Axonal atrophy with significant (P < 0.01) decrease in mean axonal, and fiber diameter was seen in the sciatic nerve section of the diabetic control group as compared to the normal control group (Figure 7b, f, and g(Fig. 7)). The TWPT with Met 250 and CHFG 400 showed statistically significant (P < 0.01) preservation of mean axonal and fiber diameter in the sciatic nerve section of the treatment group as compared to the diabetic control group (Figure 7c, e, f, and g(Fig. 7)). Whereas the TWPT with CHFG 200 showed the statistically insignificant change in mean axonal and fiber diameter in the sciatic nerve section as compared to the diabetic control group (Figure 7d, 7f, and 7g(Fig. 7)).

## Discussion

Diabetes is one of the major endocrine disorders that leads to impairment of renal and neural microvasculature (UK Prospective Diabetes Study (UKPDS), 1991[52]; Yagihashi et al., 1992[54]). Hyperglycemia-induced oxidative stress is a crucial element initiating diabetic microvascular complications (Jha et al., 2016[22]). Oxidative stress in diabetes is the result of a series of pathological events that leads to an imbalance between the pro-oxidant levels and counteracting antioxidant defense mechanisms, consequently both kidney and nerves which are an essential part of the body are susceptible to hyperglycemia-induced oxidative damage (Asieh and Mohammad, 2013[5]; Jha et al., 2016[22]).

The antioxidant effects on the diabetic kidney (Lee et al., 2008[29]) and nerves (Salma et al., 2017[44]) disorders were positively correlated with medicinal herbs supplementation. Although our previous *in vitro* study showed significant antidiabetic and antioxidant activity (Abusufyan et al., 2018[1]) to CHFG, no study was conducted to observe the effects of CHFG in diabetic renal and neural complications. We have planned our investigation to evaluate the influence of CHFG against diabetes-induced renal and neural complications.

In the current study, diabetic rats were affected by their behavioral and biochemical parameters. Diabetic rats showed severe hyperglycemia, an increase in plasma urea, uric acid, creatinine, triglyceride, and total cholesterol level. In addition, diabetic rats revealed altered behavioral parameters such as thermal hyperalgesia and cold allodynia, decreased body weight, and increased kidney weight to body weight ratio beside compromised sciatic nerve and kidney antioxidant status. Previous studies have shown that hyperglycemia can induce similar early renal and neural complications in diabetic rats (Bhaskar et al., 2014[8]; Morsy et al., 2014[36]). The administration of CHFG to the STZ induced diabetic rats improved the parameters discussed above. This protective effect of CHFG has not been reported earlier.

The CHFG showed a significant decrease in blood glucose levels at 200 mg/kg and 400 mg/kg body weight in STZ induced diabetic rats both in a single dose and in one and two weeks repeated dose treatment. Our previous study showed *in vitro* inhibition of α-glucosidase and α-amylase enzymes by CHFG extract (Abusufyan et al., 2018[1]). α-glucosidase and α-amylase are two important enzymes that convert starch to more simple sugars. Therefore, the inhibition of these enzymes reduces the rate of absorption of glucose that helps in controlling the postprandial rise in the blood glucose levels.

Neuropathic pain associated with diabetes is characterized by two important sensory symptoms such as hyperalgesia i.e. an increased response to painful stimuli, and allodynia, i.e. pain in response to a non-painful stimulus (Bianchi et al., 2012[9]). In the present study, diabetic rats showed a significant increase in paw and tail withdrawal latency at 49 °C (Thermal hyperalgesia) and tail withdrawal latency at 10 °C (Cold allodynia) as compared to the normal control group. Similar observations were also reported in earlier findings (Arun et al., 2008[4]). Administration of Met 250 and CHFG 400 resulted in a consequent decrease in both thermal hyperalgesia and cold allodynia, compared to diabetic rats. Free radical-induced oxidative stress has been implicated to play an important role in the pathogenesis of diabetic neuropathy (Nasiry et al., 2017[38]). In the present study, there was a significant increase in sciatic nerve MDA level and decrease in SOD and GSH level in diabetic rats, which is indicative of impaired oxidative balance in the sciatic nerve of diabetic rats. Treatment with Met 250 and CHFG 200 and CHFG 400 reduces oxidative stress parameters of the sciatic nerve. The anti-nociceptive effect of CHFG may be attributed to its neuroprotective effect, which might be due to its antioxidant activity. 

In the present investigation, the diabetic rats showed a significant decrease in body weight and increase in kidney weight to body weight ratio. Similar results have been found by Manikanta and Rema (2017[33]) in STZ treated four weeks of diabetic rats at a dose of 55 mg/kg, i.p. The significant decline of body weight could be due to hyperglycemia, hypo-insulinemia, loss of tissue proteins, and increased muscle wasting (Cheng and Liang, 2013[12]; Zafar and Naqvi, 2010[56]). In our experimental study, the administration of CHFG to diabetic rats significantly improved the body weight that indicates the inhibition of loss of protein and muscle tissue damage caused by hyperglycemia. CHFG also improved kidney weight to body weight ratio, which could be attributed to its protective effect against oxidative stress induced cell injury in the renal tissues.

Changes in kidney weights to body weight ratio in diabetic rats were also associated with an increase in serum urea, uric acid, and creatinine, which is considered as the marker of kidney impairment (Karahan et al., 2005[24]; Soussi et al., 2018[50]). Catabolism of protein and nucleic acid due to hyperglycemia result in the formation of non-protein nitrogenous compounds such as urea, uric acid, and creatinine in diabetic rats (Yassin and Mwafy, 2007[55]). In the present study, the diabetic rats showed an increase in the serum levels of urea, uric acid, and creatinine. The treatment with Met 250, CHFG 400, significantly attenuated the hyperglycemia-induced kidney impairment indicated above. Kidney function impairment could be attributed to oxidative damage caused by hyperglycemia (Jha et al., 2016[22]; Soussi et al., 2018[50]). Our experimental results revealed a state of oxidative stress in diabetic rats by the significant elevation in kidney MDA, decreased in SOD and GSH. Treatment with Met 250 and CHFG 400 reduces kidney MDA levels and increases the level of SOD and MDA.

The previous study revealed that diabetic patients with high cholesterol and triglyceride levels are at high risk of nephropathy and peripheral neuropathy (Aguiar et al., 2015[2]; Wheeler and Bernard, 1994[53]). In the present study, there was a significant increase in serum triglyceride and total cholesterol levels in diabetic rats as compared to normal control rats. Treatment with Met 250 and CHFG 400 reduces serum triglyceride and total cholesterol levels. This hypolipidemic activity of CHFG might also improve blood supply to the kidney and peripheral nerve, and in turn, it reduces its ischemic degeneration. The biochemical parameters were correlated with renal and neural histological studies. The histopathological observation in the kidney tissue of diabetic control rats showed an increase in glomerular volume, urinary space, and capillary congestion with hemorrhage. The previous study conducted by Malatiali et al. (2008[32]), Kartiawati et al. (2017[25]), and Krishan et al. (2017[27]) showed a similar change in typical architecture of kidney tissue of diabetic rats. Our study also reports that CHFG 400 showed marked improvement in the histological sections with a decrease in urinary space and glomeruli volume. Morphological assessments of sciatic nerve showed a decline in the mean nerve fiber and axonal fiber diameter in the sciatic nerve tissue in diabetic control rats. The study conducted by Sameni et al. (1994[45]) showed similar changes in the sciatic nerve of diabetic rats. The administration of CHFG 400 markedly reduced these changes in the sciatic nerve tissues. The findings of kidney and sciatic nerves histopathological study were in line with the biochemical parameters that revealed the beneficial role of CHFG in the treatment of diabetic renal and neural complications.

Our previous correlation study of different FG leaf extracts showed a moderately positive correlation between total flavonoids content and *in vitro* antidiabetic activity and a strong positive relationship between phenolic content, and its *in vitro* antioxidant activity (Abusufyan et al., 2018[1]). Also, quantitative phytochemical estimation of CHFG showed the presence of 352.49 mg/g quercetin equivalents (QE) of total flavonoids and 27.43 mg/g gallic acid equivalent (GAE) of total phenolic content. Characterization of CHFG by LC-MS has detected diverse phytoconstituents (Table 1(Tab. 1)), out of which 3-Methoxycatechol and p-Cymene were reported in literature for their antioxidant activity (Loo et al., 2008[30]; de Oliveira et al., 2015[14]) and Anethole was reported for its hypoglycemic, antioxidant and neuroprotective activities (Sheikh et al., 2015[47]; Bing et al., 2018[10]). Dual antidiabetic and antioxidant activity of CHFG phytoconstituents might be responsible for their protective effect against early diabetic renal and neural damage in STZ rats. 

## Conclusion

Our findings revealed a protective effect of CHFG against STZ-induced oxidative damage, alteration of biochemical parameters, and histopathology. Favorable health effects of FG leaf extracts have been documented and can partially explain the observed reno-protective and neuroprotective effects of CHFG. The current investigation, therefore, provides evidences that support the beneficial effect of CHFG against STZ-induced oxidative damage on the renal and neural system.

## Acknowledgements

The present work was partially supported by the University of Mumbai, Minor Research Grant. The authors are grateful to Dr. Abdul Razak Honnutagi, Director, Anjuman-I-Islam's Kalsekar Technical Campus, and Dr. Shariq Syed, Dean, School of Pharmacy, Anjuman-I-Islam's Kalsekar Technical Campus for providing facilities to carry out this work.

## Conflict of interest statement

The authors report no conflicts of interest associated with this manuscript.

## Figures and Tables

**Table 1:**
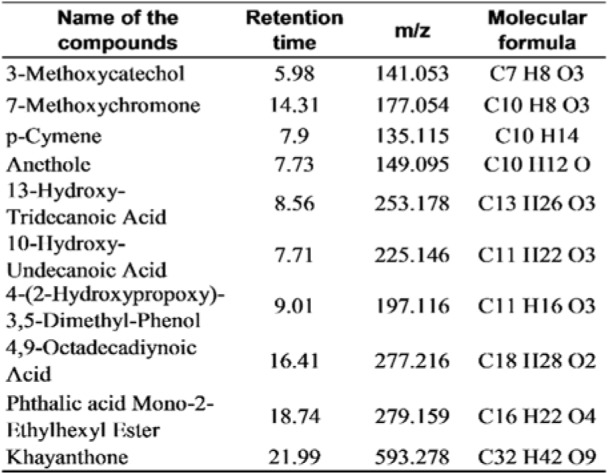
The list of the compounds detected from the CHFG by LC-MS

**Table 2:**
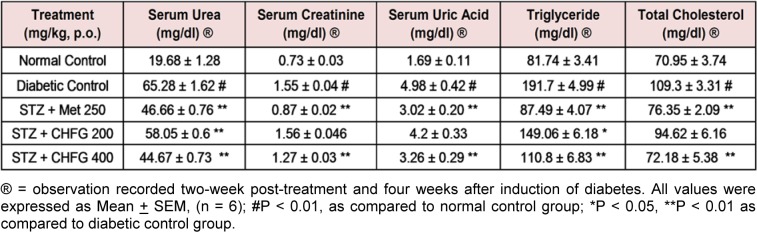
Effect of repeated-dose treatment on serum biochemical parameters in STZ-induced diabetic rats

**Table 3:**
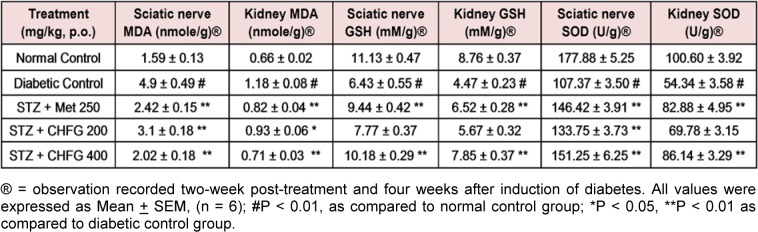
Effect of repeated-dose treatment on antioxidant activity of sciatic nerve and kidney tissue in STZ-induced diabetic rats
